# Clinical Profile and Treatment of COVID-19 Patients: Experiences from an Ethiopian Treatment Center

**DOI:** 10.4269/ajtmh.20-1356

**Published:** 2020-12-30

**Authors:** Seid Getahun Abdela, Seid Hassen Abegaz, Wondmagegn Demsiss, Koku Sisay Tamirat, Saskia van Henten, Johan van Griensven

**Affiliations:** 1 Department of Internal Medicine, College of Medicine and Health Sciences, Wollo University, Dessie, Ethiopia;; 2 Boru Meda Hospital, Dessie, Ethiopia;; 3 Department of Medical Laboratory Science, College of Medicine and Health Sciences, Wollo University, Dessie, Ethiopia;; 4 Institute of Public Health, College of Medicine and Health Sciences, University of Gondar, Gondar, Ethiopia;; 5 Institute of Tropical Medicine, Antwerp, Belgium

## Abstract

COVID-19 is not well studied in Africa. Understanding the clinical profile and management of COVID-19 will help to plan better prevention and treatment strategies taking the local context into consideration. In this study, we described the clinical profile, treatment used, and outcomes of COVID-19 patients in one of the COVID-19 treatment centers of Ethiopia, Boru Meda Hospital. An institution-based retrospective cross-sectional study was carried out using medical records of COVID-19 patients who were admitted to Boru Meda Hospital with a positive reverse transcription (RT)-PCR result from May 9, 2020 to September 20, 2020. All patients with a positive RT-PCR were admitted to the hospital, regardless of symptom and severity status. A total of 279 COVID-19 patients were included in the final analysis. The median age of patients was 28 years (interquartile range 23–40). The majority (69.5%) were male. Around a quarter (*n* = 73; 26.2%) of the patients were symptomatic, of which cough (*n* = 49; 67.1%) and fever (*n* = 32; 43.8%) were common symptoms. Among symptomatic patients, 48 (65.8%) were mild, four (5.5%) moderate, 12 (16.4%) severe, and nine (12.3%) were critical. The case fatality rate was 2.1%. Hypertension, age older than 25 years, and HIV/AIDS were significantly associated with symptomatic infection. In this study, most of the COVID-19 patients were asymptomatic. However, the proportion of severe and critical patients among those with symptoms was high. More studies are needed to assess the effect of HIV/AIDS on the severity and mortality of COVID-19.

## INTRODUCTION

In January 2020, novel SARS-CoV-2 was identified as the causative agent for a cluster of pneumonia cases initially detected in Wuhan City, Hubei Province, China.^1^ SARS-CoV-2, which causes the disease now named COVID-19, has subsequently spread throughout the world. By October 10, 2020, the virus has infected more than 37 million people and has claimed more than one million lives.^2^

The first case of COVID-19 in Ethiopia was diagnosed on March 13, 2020. As of October 10, 2020, Ethiopia recorded 82,662 cases and 1,271 deaths due to COVID-19. Since mid-January 2020, Ethiopia started to prepare for prevention and treatment of COVID-19. Different preventive measures including physical distancing were introduced, starting from the last 2 weeks of March 2020, followed by the declaration of a state of emergency with different community containment measures. In addition, treatment centers were established across different parts of the country.^3^

Until now, information about COVID-19 patients is mainly from China, Europa, and North America. There is a dearth of studies regarding COVID-19 in sub-Saharan Africa. Better understanding of the clinical and sociodemographic profiles, treatment options, and outcomes of COVID-19 patients from different parts of the world is crucial to get a full global picture.

Based on pooled reports, the outcomes of COVID-19 seem different in Africa with lower mortality.^4^ Late arrival of the disease, early preventive measures, and different sociodemographic conditions may have caused the difference. However, this is not yet supported by systematically designed studies, and the reasons causing this difference are not clear. Therefore, a better understanding of the patient profiles and outcomes in Africa is required. This will help to design better preventive and treatment strategies considering the local context. In this study, we described the clinical profile, treatment used, and outcome of COVID-19 patients in one of the COVID-19 treatment centers of Ethiopia, Boru Meda Hospital. In addition, we looked at factors associated with symptomatic cases as these groups of patients are at risk of developing severe disease.

## METHODS

### Study setting.

Boru Meda Hospital is one of the five leprosy centers in Ethiopia which was founded in 1954. On May 9, 2020, it was changed to a dedicated COVID-19 center, as a referral site of the Eastern Amhara region. It was repurposed to have different compartments, including an intensive care unit (ICU), and male and female wards. The hospital has 100 beds, five mechanical ventilators, and two continuous positive airway pressure therapy machines.

COVID-19 patients were identified through random testing of asymptomatic and symptomatic at-risk groups from bus stations, healthcare facilities, hotels, and banks. In addition, people coming from COVID-19 hot spots, contacts of confirmed cases, and clinically suspected cases were targeted in the testing strategy.

All RT-PCR SARS-CoV-2–positive cases in the region were admitted to the hospital, regardless of the severity. The initial decision to admit all patients to treatment center was because of the inconvenience of home isolation and management in many Ethiopian settings. Severe and critical cases were admitted to the ICU, whereas the others remained in the general ward. The care of COVID-19 patients is given by a multidisciplinary team composed of nurses, general practitioners, laboratory technologists, pharmacists, internists, anesthetists, and other supporting staff.

The management of COVID-19 in the center is in line with the national treatment guideline. Patients with uncomplicated upper respiratory tract viral infection and nonspecific symptoms such as fever, fatigue, cough, anorexia, malaise, muscle pain, sore throat, dyspnea, nasal congestion, or headache are considered to have a mild infection. Among adults, moderate illness is described as a patient having mild pneumonia using CURB-65 (confusion, urea, respiratory rate, and blood pressure and 65 years of age or older) and in children with non-severe pneumonia who have cough or difficulty breathing combined with tachypnea (≥ 60 breaths/minute for age < 2 months, ≥ 50 breaths/minute for age-group 2–11 months, and ≥ 40 breaths/minute for age-group 1–5 years), and no signs of severe pneumonia. Severe illness is described as a patient having severe pneumonia, acute respiratory distress syndrome, or sepsis responding to noninvasive management. In case patients do not respond to noninvasive management, or when there is respiratory failure, septic shock, and/or multiple organ dysfunction or failure, it is considered critical illness. The management of asymptomatic and mild infection is supportive. Those with moderate or severe COVID-19 receive unfractionated heparin (UFH) and dexamethasone. However, no patients were recruited in the clinical trial of experimental drug. Noninvasive and invasive ventilation is recommended for patients desaturating while on intranasal oxygen.^5^

### Study design and population.

All COVID-19 patients who were admitted to Boru Meda Hospital with positive RT-PCR from May 9, 2020 to September 20, 2020 were included in this retrospective observational study.

### Data collection procedures.

For each case, the following data were extracted from the patient medical records: sociodemographic information, symptoms, risk factors, comorbidities, COVID-19 severity classification, treatment provided, duration of hospital stay, and treatment outcomes.

### Data management and analysis.

The data extraction was performed by a trained health officer using a structured data extraction tool and entered using Epi-Data version 3.2 (EpiData Association, Odense, Denmark) and exported to SPSS version 22 (IBM, Armonk, NY). Descriptive analysis entailed the calculation of medians (interquartile range [IQR]), frequencies, and proportions (%) presented in the form of tables, graphs, and texts. To identify factors associated with symptomatic COVID-19 disease, a binary logistic regression model was fitted. Variables with a *P*-value of less than 0.2 in the bivariable analysis were entered into the multivariable analysis to control for possible confounding factors. To assess the strength of the relationship between response and independent variables, adjusted odds ratios (AORs) with 95% CIs were computed. Variables with a *P*-value of less than 0.05 in the multivariable model were considered significant factors associated with symptomatic COVID-19 disease.

### Ethical considerations.

Permission to conduct the study was obtained from Boru Meda Hospital management, Dessie, Ethiopia. Local ethics approval was secured from Ethics Committee of Wollo University, College of Medicine and Health Sciences.

## RESULTS

### Sociodemographic characteristics.

A total of 279 COVID-19 patients were included in the final analysis of the study. The median age of the patients was 28 years (IQR: 23–40), and more than one-third (35.1%) of patients were aged between 25 and 34 years. Most of the patients were male. Three were pregnant women, with a gestational age between 28 and 36 weeks, of which two were asymptomatic and one had mild symptoms (Table 1).

**Table 1:** Sociodemographic characteristics

Characteristic	Frequency (*n*)	Percentage
Age-group of the patient (years)		
Younger than 15	16	5.7
15–24	66	23.7
25–34	98	35.1
35–44	47	16.8
Older than 45	52	18.6
Gender		
Male	194	69.5
Female	85	30.5
Contact history with confirmed case		
Yes	99	35.5
No	4	1.4
Unknown	176	63.1
Occupation		
Housewife	43	15.4
Daily laborer	30	10.7
Student	31	11.1
Driver	27	9.6
Farmer	25	8.9
Healthcare worker	19	6.8
Self-employed	19	6.8
Government employed	15	5.4
Prisoner	15	5.4
Factory worker	13	4.6
Merchant	12	4.3
Others	30	10.7

### Clinical characteristics.

**Figure 1.**
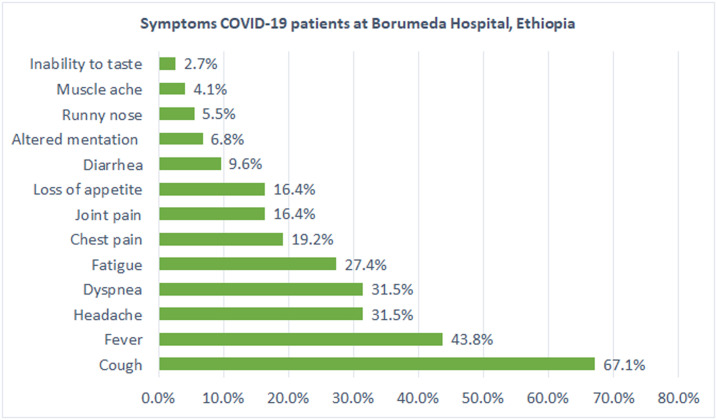
Symptoms of COVID-19 patients admitted to Boru Meda Hospital. This figure appears in color at www.ajtmh.org.

The clinical profile of patients is described in Table 2. Approximately a quarter (73; 26.2%) of patients were symptomatic, of which cough (*n* = 49; 67.1%), fever (*n* = 32; 43.8%), headache (*n* = 23; 31.5%), shortness of breath (*n* = 23; 31.5%), and fatigue (*n* = 20; 27.4%) were the most frequently reported symptoms (Figure 1). Thirty percent of symptomatic patients had a single presenting symptom, whereas 69.8% had two or more presenting symptoms; 46 (16.5%) patients had preexisting medical conditions, of which hypertension (*n* = 16; 34.7%), HIV/AIDS (*n* = 15; 32.1%), diabetes mellitus (DM) (*n* = 13; 28.2%), and asthma (*n* = 7; 15.2%) were the most common comorbidities, of which two patients had both HIV and DM, and four patients had both hypertension and DM. The median duration of illness of symptomatic patients was 5 days (IQR: 3–7). Chest X-ray was performed for only seven patients, of which five had lung infiltrates.

**Table 2:** Clinical profile

Characteristic	Frequency	Percentage
Symptomatic		
Yes	73	26.2
No	206	73.8
Severity of the disease (*n* = 73)		
Mild	48	65.8
Moderate	4	5.5
Severe	12	16.4
Critical	9	12.3
Comorbidities		
Yes	46	16.5
No	233	83.5
Comorbidities type		
Diabetes mellitus	13	28.2
Arterial hypertension	16	34.7
HIV/AIDS	15	32.1
Asthma	7	15.2
Chronic kidney disease	2	4.3
Other	2	4.3
Preadmission medication (*n* = 46)		
Angiotensin-converting enzyme inhibitors	6	13
Calcium channel blocker	8	17.4
Highly active antiretroviral viral therapy	15	32.6
Other medication	15	32.6

### Treatment.

Investigations and management of patients are described in Table 3. Unfractionated heparin was given for the majority of severe (*n* = 10; 83.3%) and all (*n* = 9; 100%) critical cases. Eighteen (6.5%) patients needed intranasal oxygen, of whom 11 (61.1%) needed a facemask for oxygen delivery; no patient was intubated. Dexamethasone 6 mg daily was given for 17 (6.1%) COVID-19 patients, for a median duration of 7 days (IQR: 5–8). Dexamethasone was given for one moderate, 10 (83.3%) severe, and six (66.7%) critical cases. The median duration of admission in the hospital was 13 days (IQR: 12–14). The majority (*n* = 271; 97.1%) of patients were discharged with improvement, six (2.1%) patients died while on treatment in the center, and the remaining two patients were transferred to other health facilities. Among the patients who died, all were male, and five were critical, one was a mild case, and four had comorbidities. A total of 10 patients were admitted to ICU and five (50%) died. Only one (2.1%) of the 48 patients who had mild disease died, whereas 55.6% (5/9) of patients with critical disease died. The median age of patients who died was 44.5 years (IQR: 40–60, range 39–60).

**Table 3:** Investigations and management

Characteristic	Frequency	Percentage
Complete blood cell count		
Yes	24	8.6
No	255	91.4
White blood cell count (*n* = 24) (cells/µL)		
Leukopenia (< 4,000)	2	8.7
Normal (4,000–10,000)	12	47.8
Leukocytosis (> 10,000)	10	43.5
Lymphocyte count (*n* = 24)		
< 1,500	14	58.3
≥ 1,500	10	41.7
Treatment given		
Unfractionated heparin	22	7.9
Antibiotics	38	13.6
Intranasal oxygen	18	6.4
Dexamethasone	17	6.1

### Factors associated with symptomatic COVID-19.

In the bivariable logistic regression analysis, patient’s age, presence of hypertension, and HIV/AIDS coinfection were factors associated with symptomatic COVID-19 disease (Table 4). These factors remained statistically significant in the multivariable analysis, except for diabetes mellitus. Patients aged 25–34 years (AOR: 2.68; 95% CI: 1.10–6.51), 35–44 years (AOR: 3.85; 95% CI: 1.41–10.52), and older than 45 years (AOR: 7.67; 95% CI: 2.50–20.03) were more likely to have symptomatic COVID-19 disease than those younger than 24 years, respectively. For patients with hypertension, the odds of symptomatic COVID-19 was 4.61 (95% CI: 1.32–16.09) times higher than that for individuals with no hypertension. Similarly, patients with HIV coinfection were also more likely to be symptomatic (AOR: 3.27; 95% CI: 1.03–10.33).

**Table 4:** Factors associated with symptomatic COVID-19

Variable	Symptomatic COVID-19		Crude OR	Adjusted OR
	Yes	No		
Age-group of the patient (years)				
Younger than 24	8	74	1	1
25–34	23	75	2.83 (1.19–6.74)	2.68 (1.10–6.51)*
35–44	14	33	3.92 (1.50–10.25)	3.85 (1.41–10.52)*
Older than 45	28	24	10.79 (4.34–26.82)	7.67 (2.50–20.03)*
Female patient	18	67	0.67 (0.37–1.24)	0.51 (0.25–1.04)
Contact history with confirmed cases	20	79	1.64 (0.92–2.96)	1.25 (0.63–2.49)
Diabetes mellitus	9	4	7.10 (2.11–23.85)	2.23 (0.55–8.94)
Hypertension	12	4	9.93 (3.09–31.92)	5.26 (1.50–18.46)*
HIV coinfection	8	7	3.49 (1.22–10.02)	3.27 (1.03–10.33)*

OR = odds ratio.

* *P*-value less than 0.05.

## DISCUSSION

The world is generating evidence regarding COVID-19 rapidly. This has helped to understand the clinical profile and improve management of the disease. The severity and mortality of COVID-19 depends on several sociodemographic and clinical parameters which may differ per area. However, there is a paucity of evidence about African COVID-19. In this study, we described the clinical profile and treatment of all RT-PCR–positive cases in one of the treatment centers of Ethiopia.

Most of our SARS-COV-2–infected patients were asymptomatic. This is much higher than that reported in other studies, which mostly identified cases through population-based testing.^6,7^ As most of these studies did not do longitudinal follow-up to assess for symptom development, it is likely that the proportion of patients who remain asymptomatic is even lower than that was reported. In our case, patients were admitted to hospital, and daily symptom assessment was performed by clinicians working in the COVID-19 treatment center, and therefore, we can confidently say they remained asymptomatic. The high percentage of asymptomatic patients may be explained by a large proportion of young patients. The testing strategy, which focused on at-risk populations with a high number of human contacts regardless of complaints, may also have caused this finding. In addition, the possibility of false-positive result cannot be ruled out. The role of asymptomatic individuals in disease transmission needs further studies.

More than two-thirds of symptomatic patients had cough. This is much higher than that the large cohort CDC reported in the United States.^8^ Whereas the percentage of symptomatic patients having fever, dyspnea, and headache was similar to that of other studies carried out in China and the United States, diarrhea, taste disturbances, and muscle pain were less common in this study.^8–10^ Using fever as entry point for diagnosis of COVID-19 may be misleading as only less than half of symptomatic patients were febrile from this cohort. We stress here the importance of considering COVID-19 even in the absence of recorded fever and other compatible symptoms.

Among symptomatic patients, nearly two-thirds had mild disease, which is less than the 80% recorded in the CDC report from China and comparable with results from the Democratic Republic of the Congo (DRC).^11,12^ Although the proportion of severe and critical illness was higher in our population, the overall case fatality rate was comparable to the China CDC report but much lower than that reported in the DRC study. This can be explained by difference in the admission criteria. However, unlike other reports, we had one death from a noncritical group with unidentified cause of death.^11^ Diabetes mellitus, arterial hypertension, and HIV/AIDS were common comorbidities. As age increased older than 25 years, the odds of being symptomatic also increased, with patients older than 45 years having the highest risk. Likewise, arterial hypertension and HIV/AIDS infection increased the odds of symptomatic disease. A study from South Africa also showed an association of HIV/AIDS infection with COVID-19 mortality, although results showed confounding bias may be an issue.^13^ Further studies are needed to assess the effect of HIV/AIDS on the severity and mortality of COVID-19.

Most of the moderate, severe, and critical COVID-19 cases received intravenous antibiotics prophylactically, because diagnosing superimposed bacterial infections in this setting is difficult. Similarly, dexamethasone was given for most of the severe and critical cases, with one moderate patient also receiving dexamethasone. This is of particular concern as extrapolating evidence from Western settings may not work for our population where HIV/AIDS infection is quite common. In the face of already impaired immunity, this group of patients may be at increased risk of side effects. The use of UFH for severe and critical cases is consistent with recommendations from expert societies.^14^ However, it was impossible to determine the baseline and follow-up coagulation profile in the study setting, and therefore, unwanted side effects of heparin including supratherapeutic coagulation profiles and heparin-induced thrombocytopenia could not be monitored.

This study has several strengths. First, it was possible to look the clinical profile of all patients with positive RT-PCR for SARS-CoV-2 unlike other settings where only symptomatic cases are admitted. Second, symptom evaluation and severity assessment were performed in the hospital daily by treating clinicians. The limitations of the study include the lack of thorough investigations including chemistry and the coagulation profile, which made the clinical profile assessment incomplete. In addition, it was not possible to determine factors associated with severe disease and death because these events were rare.

In this study, most of the COVID-19 patients were asymptomatic, with an overall case fatality ratio of 2.1% and a median age of 28 years. Older age, HIV/AIDS, and arterial hypertension were independently associated with symptomatic COVID-19. The routine use of intravenous antibiotics for COVID-19 patients may impose the risk of unwanted side effects and can fuel antibiotic resistance. We stress the importance of developing clinical and laboratory criteria for the diagnosis of bacterial superinfection. Studies are urgently needed to see the effect of HIV/AIDS on the severity and mortality of the disease and the role of dexamethasone in decreasing mortality.
